# Mechanotransduction Failure and Molecular Rescue in Gastric Cancer: Kinetotherapy Across the IL-6/STAT3–Myostatin/ACVR2B–Akt/mTOR Axis

**DOI:** 10.3390/medsci14030365

**Published:** 2026-07-01

**Authors:** Stefan Oprea, Adrian Vasile Dumitru, Dan Dumitrescu, Maria Fulina, Matei Șerban, Răzvan-Adrian Covache-Busuioc, Corneliu Toader, Lucian Eva

**Affiliations:** 1 Faculty of General Medicine, “Carol Davila” University of Medicine and Pharmacy, 050474 Bucharest, Romaniamateiserban@innbn.com (M.Ș.);; 2 Department of Anatomy, “Carol Davila” University of Medicine and Pharmacy, 050474 Bucharest, Romania; 3 Puls Med Association, 051885 Bucharest, Romania; 4 Department of Pathology, Faculty of Medicine, “Carol Davila” University of Medicine and Pharmacy, 030167 Bucharest, Romania; 5 County Clinical Emergency Hospital “Sf. Ap. Andrei”, 900591 Constanța, Romania; maria.fulina@yahoo.com; 6 Department of Neurosurgery, “Carol Davila” University of Medicine and Pharmacy, 050474 Bucharest, Romania; 7 Department of Vascular Neurosurgery, National Institute of Neurology and Neurovascular Diseases, 077160 Bucharest, Romania; 8 ”Nicolae Oblu” Clinical Hospital, 700309 Iasi, Romania

**Keywords:** gastric cancer, cancer cachexia, kinetotherapy, mechanotransduction, myostatin, calcium signaling, mitochondrial dysfunction, PI3K/Akt/mTOR

## Abstract

Muscle wasting associated with gastric cancer represents a complex, multifactorial systems disorder involving inflammatory, anabolic, mechanosensory, calcium-regulatory, mitochondrial, and proteostatic disruption. This review synthesizes current evidence regarding the cellular and physiological mechanisms involved in skeletal muscle dysfunction in gastric cancer and provides a unifying framework centered on loss of signaling coherence. Specifically, it examines IL-6/STAT3 and NF-κB inflammatory signaling, the myostatin–activin–ACVR2B–SMAD pathway, PI3K/Akt/mTOR signaling, mechanotransduction, excitation–metabolism coupling, calcium homeostasis, mitochondrial function, and proteostasis. Although individual components of these pathways have been implicated in muscle wasting associated with chronic disease, current evidence suggests that they interact through positive feedback loops. Inflammation, anabolic resistance, impaired force-to-signal conversion, mitochondrial stress, altered intracellular calcium homeostasis, and disrupted protein quality control may reinforce one another, contributing to metabolic, structural, and transcriptional instability. Within this context, muscle wasting reflects not only loss of muscle mass or strength, but also loss of functional integrity resulting from disrupted integration of mechanical, metabolic, inflammatory, and anabolic signals. Given the systemic nature of these effects, this review proposes kinesitherapy as a potentially useful nonpharmacological adjunctive strategy that may modulate inflammation, restore responsiveness to mechanical stimuli, support calcium homeostasis and mitochondrial function, improve anabolic sensitivity, and maintain protein quality control. Overall, this review presents a systems-biology model of gastric cancer-associated muscle wasting and supports further investigation of exercise-based therapies for this condition.

## 1. Introduction

Skeletal muscle is not simply a contracting tissue, but rather an extremely sensitive biological system that can respond to various types of information—mechanical, hormonal/endocrine, metabolic, inflammatory/biochemical, and bioenergetic [1]. Therefore, muscle dysfunction associated with gastric cancer should not only be viewed as a decrease in the amount of muscle mass or rate of protein synthesis, but as a gradual deterioration of the signal integration and coordination required for adaptation, repair, and recovery of normal muscle function [2,3].

Muscle wasting is used in this review to describe the progressive reduction in the size and quality of skeletal muscle and in its ability to perform functional activities, which occurs due to systemic stress caused by cancer. Muscle wasting is closely related to other terms—sarcopenia, myopenia, malnutrition, and cachexia—but they are not synonymous. Generally speaking, sarcopenia describes decreased muscle strength, performance, and mass, usually seen during aging or in chronic diseases [4]. Myopenia typically refers to low muscle mass. Imaging technologies provide common ways of measuring muscle mass. Malnutrition represents poor dietary intake, absorption, or utilization. Cachexia describes a syndrome caused by cancer, resulting in continued loss of skeletal muscle with or without concurrent fat loss [5]. Nutritional supplementation does not always reverse cachexia. In patients with gastric cancer, the above terms may overlap, but muscle wasting specifically addresses the structural depletion of skeletal muscle along with an inability of muscle cells to effectively respond to biological signals, including anabolic resistance, mitochondrial dysfunction, enhanced proteolytic activity, decreased mechanoadaptability, and diminished functional capacity [6].

One of the most significant biological triggers for muscle wasting is systemic inflammation. Gastric cancer can lead to continuous release of pro-inflammatory cytokines such as interleukin-6 (IL-6), interleukin-8 (IL-8), and tumor necrosis factor-alpha (TNF-alpha). IL-6 has been identified as critical, since it activates the Janus kinase/signal transducer and activator of transcription 3 pathway, commonly referred to as JAK/STAT3 [7]. Briefly, when IL-6 binds to its receptor complex on the cell surface, it causes the activation of JAK kinases and subsequently leads to the phosphorylation of STAT3. Once STAT3 has been phosphorylated, it moves to the nucleus, where it controls the expression of genes involved in inflammation, proteolysis, mitochondrial dysfunction, and ultimately muscle catabolism [8]. Acute STAT3 activation may facilitate immunoregulatory functions and/or tissue repair; however, prolonged STAT3 activation will likely cause skeletal muscle to become less capable of responding adaptively to stress and instead enter a catabolic state. TNF-alpha functions via nuclear factor kappa B (NF-kappa B), another key inflammatory signaling cascade. NF-kappa B activation results in the generation of long-lasting inflammatory/proteolytic signals and mitochondrial dysfunction. Moreover, IL-6/STAT3 and TNF-alpha/NF-kappa B act together and interact with redox signaling, mitochondrial bioenergetics, and protein degradation systems. For example, STAT3-dependent mitochondrial alterations may produce reactive oxygen species (ROS), whereas NF-kappa B signaling may create a self-reinforcing loop enhancing inflammatory responses. Collectively, these interactions help clarify how nutritional support alone cannot easily reverse muscle loss in gastric cancer [9,10].

Additionally, gastric cancer-related muscle wasting involves chronic repression of anabolic signaling. The myostatin–activin–ACVR2B pathway is a primary mechanism underlying this phenomenon. Activated by binding to their specific type II receptors, particularly ACVR2B, myostatin and activin A activate SMAD2/3-dependent transcriptional programs. These programs inhibit myogenic differentiation, reduce responsiveness to typical anabolic stimuli, and work synergistically with FOXO transcription factors to induce ubiquitin–proteasome-mediated degradation via downstream effector proteins such as MuRF1 and atrogin-1 [11,12].

Hence, skeletal muscle experiences two forms of biological impact: degradation is enhanced, while the molecular machinery for repair/growth/recovery is compromised. These disruptions occur within a larger network of endocrine/metabolic elements, including tumor-derived substances, adipokines/myokines, dysfunctional insulin signaling, reduced food intake, treatment-induced stress, and systemic inflammation [13,14]. Consequently, skeletal muscle plays a pivotal role in the multi-tissue communication network linking tumor biology, host metabolism, immune stimulation, substrate utilization, and whole-body energy homeostasis. If this network fails, then muscle enters into a state of anabolic resistance; i.e., previously adaptive stimuli such as amino acids, insulin, mechanical load, and rehabilitation elicit significantly weaker biological responses [15]. Failure in mechanotransduction represents an additional—and perhaps underappreciated—contributor to gastric cancer-related muscle wasting. Mechanotransduction enables skeletal muscle to transform mechanical stresses imposed by movement/loading into biochemical signals via numerous intermediate steps, including integrins, costameres, mechanosensitive ion channels, calcium-dependent signaling, cytoskeletal networks, and mechanosensitive transcriptional programs [16,17]. Physiological application of mechanical stresses allows for translation of mechanical stresses into anabolic signals, mitochondrial adaptations, structural remodeling, and preservation of contractile function. However, in gastric cancer, systemic inflammation, redox stress, dysmetabolism, nutritional deficiencies, treatment burden, and decreased physical activity may impair this conversion from force to signal [18,19]. Thus, muscle fibers may receive mechanical stresses but fail to convert these stresses into coordinated adaptive responses.

The biological pathways that contribute to gastric cancer-related muscle wasting are thus highly interconnected. Continuous inflammation, anabolic resistance, calcium dyshomeostasis, mitochondrial dysfunction, proteostasis imbalance, and disruption in mechanotransduction mutually reinforce one another through positive feedback mechanisms. Abnormal mitochondria generate ROS; ROS damage calcium-handling proteins; abnormal calcium signaling promotes proteolysis; proteolytic remodeling alters cytoskeletal and costameric structure; and impaired costamere integrity diminishes mechanotransduction and anabolic signaling [20]. Instead of being independent mechanisms, these processes represent part of a spectrum of progressive disorganization of signaling coherence at molecular, organellar, cellular, tissue, and systemic levels. From this perspective, gastric cancer-related muscle wasting may be conceptualized as a multifaceted failure in signaling coherence that involves inflammatory activation, endocrine disruption, organelle dysfunction, transcriptional reprogramming, maladaptive responses at the tissue level, and impaired inter-organ communication [21].

This framework also creates a logical rationale for reevaluating the role of kinetotherapy in rehabilitation as more than just supplemental. Structured clinical supervision with therapeutic movements and exercises are included when describing kinetotherapy. The structured, clinically supervised interventions are tailored to the individual’s onco-histological diagnosis, nutritional reserve, degree of inflammation, fatigue levels, current treatment regimen, current state of recovery from surgery, and functional abilities [22]. Kinetotherapy therefore includes much more than unsupervised physical activity; it consists of a repetitive mechanically-physiologically-metabolically stimulated input into skeletal muscle via supervized contractions and loads. There is a large amount of evidence indicating that gastric cancer associated muscle wasting results from disruptions in several distinct yet connected regulatory systems (e.g., inflammatory responses) rather than one specific pathway [23]. Repetitive contractile inputs, as well as repetitive loads placed upon muscles can result in modulation of the aforementioned pathways and processes including inflammatory response mechanisms, intracellular calcium concentrations, mechanosensory transduction pathways, mitochondrial biogenesis, insulin sensitivity, proteostasis and anabolic signals through mechanisms involving Akt/mTOR [24,25,26].

From this perspective, kinetotherapy may be interpreted as a systems-level biological input capable of partially restoring signaling coherence across several disrupted networks involved in gastric cancer-associated muscle wasting.

The goal of this review article was to synthesize current understanding regarding muscle wasting due to gastric cancer into a mechanistic framework that incorporates inflammatory signaling, endocrine/metabolic dysregulation, mechanotransductive failure, calcium dyshomeostasis, mitochondrial dysfunction, and proteostatic collapse. Special emphasis was placed upon the possibility that kinetotherapy may restore—at least in part—coherence in signaling among these interconnected biological systems. In this model, skeletal muscle is not passively affected by gastric cancer. Skeletal muscle is actively engaged in integrating stress caused by gastric cancer, adapting to host needs, and contributing to therapeutic responsiveness.

## 2. The Myostatin–ACVR2B–SMAD Axis and the Suppression of Anabolic Competence

### 2.1. Ligand–Receptor Dynamics: Myostatin, Activin A, and ACVR2B-Dependent Transcriptional Control

Although myostatin–activin receptor signaling provides essential input in determining the degree of hypertrophic response of skeletal muscle to external stimuli, it also plays a major role in determining when a muscle transitions from a highly responsive and adaptable tissue to a transcriptionally restrictive and catabolically permissive one. The TGF-β family cytokine myostatin (GDF-8), together with activin A, binds to the type II activin receptor ACVR2B, a transmembrane serine/threonine kinase receptor primarily found in skeletal muscle. When activated by its ligands, ACVR2B dimerizes with either ALK4 or ALK5, which function as type I receptors. Once formed, the dimerized ACVR2B and type I receptors lead to phosphorylation of SMAD2 and SMAD3 at the SSXS motif [27]. Upon phosphorylation, the SMADs combine with SMAD4 and travel to the nucleus, where they interact with chromatin-modifying enzymes and co-transcriptional regulatory molecules to suppress transcription of myogenic genes, satellite cell activation, and protein synthesis [28,29].

Substantial information regarding the biological significance of this pathway has emerged from examination of sera collected from patients with cachexia secondary to gastric cancer. Increased serum levels of IL-6, IL-8, FABP3, and FSTL-1 were observed. Conversely, decreased leptin levels were noted. Increased myostatin and IL-15 mRNA expression was also identified in the gastric tumors examined. However, comparative portal and peripheral venous sampling reveals that these factors are not merely products of the tumor itself, but represent a comprehensive endocrine relationship between tumor and host tissues. Host tissue involvement includes possible participation from skeletal muscle in creating and sustaining the signaling environment [30]. Therefore, while there is clearly a contribution to ACVR2B activation from tumor-derived factors, the total signaling environment appears to develop through continuous bidirectional communication between the tumor and host tissues. Chronic ligand availability may therefore maintain persistent SMAD2/3 activation and decrease anabolic transcriptional capability [31].

**Figure 1:**
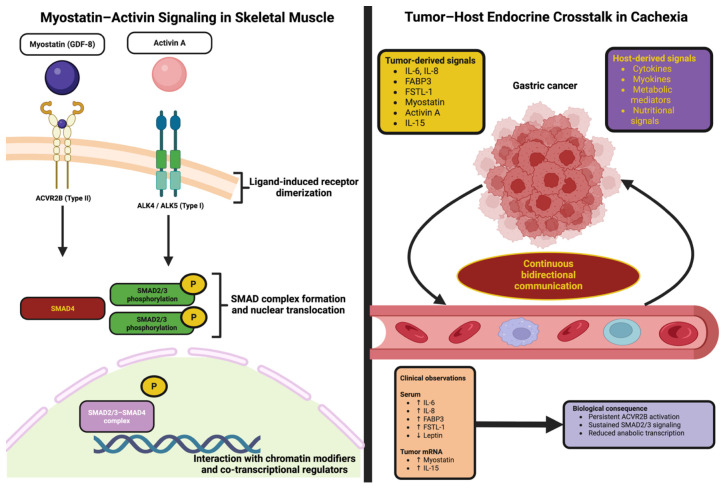
Myostatin and activin A activate ACVR2B–ALK4/5 signaling, leading to SMAD2/3 phosphorylation, SMAD4-mediated nuclear translocation, and reduced anabolic transcription. In gastric cancer cachexia, bidirectional tumor–host signaling sustains this pathway and contributes to impaired skeletal muscle adaptability.

In addition to influencing myofiber size, ACVR2B signaling may impact the environment necessary for maintaining structural and metabolic stability of skeletal muscle. Increasing data indicate that activin A can disrupt vascular homeostasis via decreases in PGC-1α activity and endothelial dysfunction. Vascular disruptions such as these may lead to reduced capillary density and tissue perfusion. As a result, delivery of oxygen and nutrients to metabolically active muscle tissue may become inadequate, leading to diminished anabolic capabilities [27]. Given that many patients diagnosed with gastric cancer exhibit systemic inflammation, anemia, malnutrition, and decreased physical activity, additional disruptions to skeletal muscle homeostasis may occur. Overall, these findings support the view that the myostatin–activin signaling axis acts not only as a regulator of intracellular transcriptional programming, but also as an environmental modulator impacting the structural and metabolic environments in which skeletal muscle maintains function and adaptability [32]. To integrate these mechanistic and systemic observations, Figure 1 summarizes how myostatin–activin signaling links ACVR2B–SMAD transcriptional repression with tumor–host endocrine crosstalk in gastric cancer cachexia.

### 2.2. Crosstalk with PI3K/Akt/mTOR: Erosion of Translational Capacity and Ribosomal Drive

An understanding of the functional effects of SMAD signaling is best achieved in the context of PI3K/Akt/mTOR, the predominant anabolic signaling axis supporting skeletal muscle growth and maintenance. Briefly, activation of insulin and IGF-1 receptors triggers PI3K activation through phosphorylation of IRS-1. Subsequent activation of Akt occurs after the production of phosphatidylinositol (3,4,5)-trisphosphate causes phosphorylation of Akt at Thr308 and Ser473. Activation of mTORC1 by Akt enables phosphorylation of p70S6K and 4E-BP1, leading to increased ribosomal biogenesis and translation initiation [33].

Under normal physiological conditions, this pathway enables skeletal muscle to convert nutritional, hormonal, and mechanical stimuli into protein synthesis and adaptive growth. Continuous myostatin–ACVR2B signaling impairs this anabolic cascade. Reduced Akt phosphorylation has been associated with SMAD2/3 activation via direct transcriptional regulation or indirect modification of upstream effectors, including degradation of IRS-1 and activation of phosphatases. Diminished Akt activity limits mTORC1’s ability to support protein synthesis, resulting in decreased phosphorylation of p70S6K and preventing eIF4E from being released from 4E-BP1. The translation apparatus remains structurally intact; however, it becomes functionally less capable of supporting protein synthesis, resulting in what is commonly referred to as anabolic resistance [34].

These impairments in anabolic signaling have implications extending beyond molecular biology. Clinical relevance is demonstrated by observations that gastric cancer patients with reduced skeletal muscle mass prior to receiving chemotherapy experience greater adverse effects from chemotherapy treatments and worse survival rates. Neoadjuvant therapies also accelerate loss of muscle mass and functional capacity. Together, these observations suggest that compromised PI3K/Akt/mTOR competence reduces skeletal muscle’s ability to recover from sequential episodes of metabolic insult and inflammation, thus making it increasingly vulnerable to adverse effects caused by subsequent stressors [35]. High-resolution RNA sequencing studies investigating the molecular heterogeneity of cancer cachexia have provided insight into differences in the degrees of translational suppression, fiber-type atrophy, and metabolic dysfunction among different transcriptional subgroups. Such heterogeneity implies that mTOR pathway impairment exists along a continuum, thus suggesting that future therapeutic strategies may benefit from molecular characterization for improved precision in patient stratification and treatment planning [36].

Functionally, it appears that SMAD signaling alters signaling balance rather than completely eliminating anabolic signaling. While residual Akt/mTOR activity remains present, it is generally not sufficient to counteract ongoing proteolysis. This conceptualization is clinically relevant since it implies that partial restoration of Akt/mTOR activity may improve net protein balance, thus providing a basis for developing therapeutic interventions aimed at restoring anabolic signaling [37]. For reference purposes, Table 1 lists primary signaling pathways implicated in cancer-induced muscle wasting, highlighting points of convergence, functional impacts, and areas for therapeutic intervention.

### 2.3. FOXO-Driven Proteolytic Priming: Integration of SMAD Signaling with Ubiquitin–Proteasome and Autophagy Systems

Suppression of protein synthesis limits skeletal muscle’s capacity for new protein synthesis, while activation of proteolytic processes ensures continued degradation of pre-existing proteins. Primary regulators of catabolic gene expression are the Forkhead box O (FOXO) transcription factors, namely FOXO1 and FOXO3. After dephosphorylation, FOXO transcription factors dissociate from cytosolic retention complexes, translocate to the nucleus, and regulate transcriptional programs involved in ubiquitin-mediated proteolysis and autophagy [50]. Notably, two E3 ubiquitin ligases responsible for tagging structural proteins, including myosin heavy chain, for destruction through the 26S proteasome are important downstream targets: MuRF1/TRIM63 and atrogin-1/FBXO32. Concurrently with this increase in ubiquitin-mediated proteolysis, FOXO induction increases transcription of autophagy-related genes, namely ULK1, LC3, and BNIP3, facilitating autophagosome formation and selective removal of damaged mitochondria through mitophagy. Initially, these pathways function as adaptive mechanisms ensuring quality control during stressful conditions. However, prolonged activation leads to a shift toward net structural and functional loss of skeletal muscle [51].

Critical to the understanding of the biochemical interactions between SMAD signaling pathways and FOXO pathways is recognition that these two pathways converge at multiple levels of transcriptional regulation, generating an environment conducive to protein degradation. Specifically, SMAD-mediated repression of Akt signaling contributes to increased FOXO activation. Furthermore, SMAD-containing transcription complexes may directly or indirectly upregulate atrophy-related genes [52]. Recent single-nucleus multi-omics studies have demonstrated that cancer cachexia is characterized by widespread transcriptional programs similar to those seen with denervation, including overexpression of myogenin and related regulatory factors. These data collectively provide evidence that muscle wasting represents a broader reprogramming of cellular identity rather than isolated activation of discrete catabolic pathways [36,53,54]. Studies conducted using animal models have shown that inhibition of several components within this network, including myostatin and myogenin, resulted in a reduction of muscle atrophy. Importantly, these studies highlight the necessity to develop predictive molecular signatures of muscle wasting to enhance the development and implementation of specific therapeutic strategies and patient stratification [34].

Furthermore, the interplay among these signaling networks may clarify why nutritional supplementation alone fails to reverse progressive muscle wasting in patients with gastric cancer despite adequate caloric consumption. Cachectic states appear to create a catabolically primed state in skeletal muscle, such that degradative pathways remain sensitive to subsequent stimuli. Systemic inflammation and metabolic derangements may interact with cachexia-induced muscle wasting to produce self-perpetuating cycles of tissue loss. As such, successful therapeutic strategies will likely involve suppression of proteolytic signals together with restoration of anabolic responsiveness [55].

Therefore, kinetotherapy is a plausible therapeutic strategy for enhancing skeletal muscle function. Mechanically stimulated skeletal muscles can activate Akt signaling, modulate inflammatory pathways, and affect transcriptional regulators controlling muscle adaptation and recovery processes. Kinetotherapy may stimulate multiple elements within the catabolic network simultaneously rather than addressing a single component individually [56]. The combination of the myostatin-activin-ACVR2B-SMAD pathway, the decrease in PI3K/Akt/mTOR signaling and FOXO-driven proteolytic priming indicate the switch from anabolic to catabolic muscle processes that occur due to muscle wasting caused by gastric cancer. Muscle function therefore results from specific tumor-host interaction factors such as gastric cancer-related hormonal signals, inflammatory reactions, malnutrition, and limited adaptability due to therapy. However, if skeletal muscle can react to external mechanical stimuli through conversion of these stimuli into biochemical signals, anabolic potential can then be regained. As a consequence, the next section will provide information on mechanotransduction as a connection point between the molecular mechanisms already discussed (the myostatin-activin-ACVR2B-SMAD pathway; PI3K/Akt/mTOR signaling; FOXO driven proteolytic priming), and the biological base for kinetotherapy.

## 3. Mechanotransduction Pathways in Skeletal Muscle: From Mechanical Load to Gene Expression

### 3.1. Integrin–Costamere Signaling and Mechano-Structural Continuity

Muscle’s adaptability requires the transformation of mechanically activated forces into chemical signals. Force is converted into chemically active signals through the costamere. The costamere is a mechanically sensitive structure composed of a series of macromolecules that connect the sarcomere with the extracellular matrix. As such, costameres enable the transmission of force across the sarcolemma while functioning as signaling hubs. The costamere contains α7β1 integrin dimers, dystrophin-associated glycoproteins, talin, vinculin, paxillin, and a variety of adapter molecules. Collectively, the costamere constitutes a dual mechanical and biochemical interface that transforms externally applied loads into intracellular signaling activity [57,58].

Force application results in the conformational activation and aggregation of integrins, which in turn recruits focal adhesion kinase (FAK). Following tyrosine phosphorylation at Tyr397, FAK serves as a scaffold for Src-family kinases, p130Cas, and other signaling intermediary molecules. This mechanochemical platform initiates a number of downstream pathways, including the Ras–Raf–MEK–ERK pathway, RhoA/ROCK-mediated cytoskeletal reorganization, and phosphoinositide-based pathways that interact with anabolic signaling pathways [59]. Costamere integrity is critical because it allows mechanical information to be moved not only across the sarcolemma, but also through actin filaments and desmin intermediate filaments to the nucleus. Once at the nucleus, this mechanical information can influence many aspects of nuclear function, including nuclear positioning, nuclear morphology, chromatin organization, and transcriptional activity [60].

Therefore, mechanotransduction should be viewed as a physically continuous biochemical signaling system that extends from the ECM to the genome. Gastric cancer may interfere with this system at various points. An abnormal ECM composition, i.e., altered interaction with laminin or decreased intermolecular crosslinking of collagen, may result in disrupted integrin aggregation and costamere dynamics. Simultaneously, ROS may oxidatively modify cytoskeletal proteins, thereby reducing cytoskeletal elasticity and decreasing the efficiency with which force is transmitted [61]. Although these changes do not necessarily mean that mechanotransduction ceases entirely, they can decrease the degree to which mechanical information is accurately translated into biochemical signals. Additionally, because anabolic suppression limits compensatory mechanisms necessary for maintaining structural homeostasis of muscle tissue, these disruptions may be compounded in patients with gastric cancer [62].

### 3.2. Mechanosensitive Ion Channels and Calcium-Encoded Signal Fidelity

In addition to membrane-based signaling using receptors and cytoskeletal-based signaling using actomyosin contraction, skeletal muscle uses another method to convert mechanical forces into chemical signals. This method utilizes mechanically sensitive ion channels embedded within the sarcolemmal membrane that translate mechanical deformation of the membrane into intracellular ionic signals. These ionic signals encode both the magnitude and type of mechanical stimulus applied to the cells. Among these channels, Piezo1 represents a particularly interesting candidate, since it functions as a primary mechanosensor. During mechanical stimulation, Piezo1 detects plasma membrane deformation, permitting Ca^2+^ and possibly other cations to enter the cell [16]. Additional mechanosensitive channels, such as TRPV4 and TRPC1, are responsible for supporting Ca^2+^ entry into cells during repeated or prolonged mechanical stimulation. These channels are found in high mechanical-strain areas near myofibrillar bundles and in locations subjected to significant global cellular deformation [63].

Calcium entry is an extremely fast way to respond to mechanical stimulation. However, the biological importance of calcium entry is greater than simply providing an influx of ions. Instead, mechanical information is encoded by varying patterns of calcium signaling characterized by differences in amplitude, frequency, duration, and location. Intracellular free calcium elevation occurring locally generates calcium microdomains that activate calmodulin-dependent pathways. Of most importance among these pathways are Ca^2+^/calmodulin-dependent protein kinase II (CaMKII) and calcineurin. Both CaMKII and calcineurin have distinct responses to different temporal patterns of intracellular calcium oscillation [64]. Calcineurin generally responds to sustained, low-amplitude intracellular calcium elevation, promoting NFAT translocation into the nucleus and regulating genes involved in oxidative metabolism, angiogenesis, and fiber-type specification [65]. By contrast, CaMKII primarily responds to high-frequency transient increases in intracellular calcium, activating transcription through phosphorylation of CREB and related transcription factors. Therefore, calcium acts as a second messenger converting mechanical stimuli into specific transcriptional responses. There are numerous ways in which gastric cancer may disrupt this calcium-encoding system. Lipid peroxidation products of membrane lipids can affect channel sensitivity to mechanical deformation. Direct oxidation of channel proteins can alter their electrical properties. Altered transcriptional regulation of channel subunit mRNAs by inflammatory signaling can alter both expression levels and subcellular localization of channels. Similarly, disruption of cytoskeletal–sarcolemmal coupling can limit force transmission to mechanosensitive channels despite continued application of mechanical stimuli [66].

**Figure 2:**
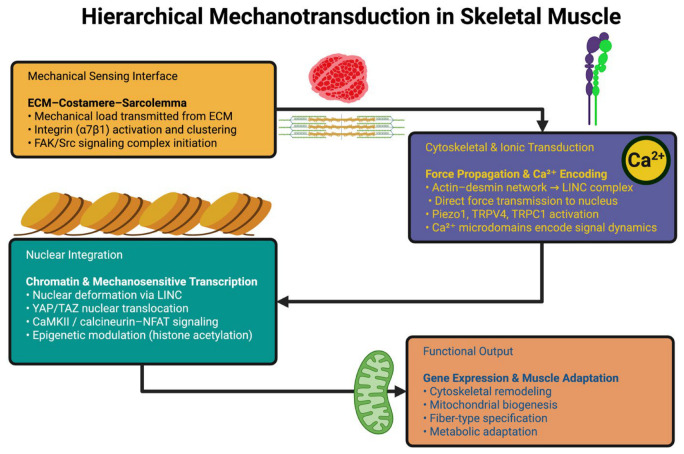
Skeletal muscle mechanotransduction is depicted as a hierarchical cascade connecting extracellular mechanical load to gene expression. Mechanical forces are sensed at the ECM–costamere–sarcolemma interface via integrin–FAK signaling, transmitted through cytoskeletal networks and mechanosensitive ion channels encoding signals as Ca^2+^ dynamics, integrated at the nuclear level through LINC-mediated deformation and YAP/TAZ- and Ca^2+^-dependent pathways, and ultimately leading to gene expression programs regulating structural remodeling, metabolism, and fiber-type adaptation.

While these changes may not completely abolish calcium signaling, they can greatly reduce the fidelity with which mechanical information is encoded. Furthermore, impaired calcium signal fidelity can have implications for metabolism and impact a muscle’s ability to satisfy changing energy demands [67]. Mechanosensitive ion channels serve as a significant intersection point between mechanical, metabolic, and biochemical signals. Dysfunction of these channels may therefore significantly contribute to muscle wasting secondary to gastric cancer [68]. Figure 2 demonstrates this hierarchical relationship, illustrating how mechanical forces are converted into biochemical signals using membrane-based, cytoskeletal-based, ionic-based, and nuclear-based mechanisms.

### 3.3. Nuclear Mechanotransduction, Chromatin Dynamics, and Activity-Dependent Transcription

Finally, these mechanotransductive processes converge at the nucleus. Cytoskeleton-generated forces are transmitted to the nuclear lamina through the LINC complex, thereby influencing chromatin structure and gene expression. Changes in nuclear deformation can directly influence gene expression through alterations in chromatin accessibility, chromatin structure, and/or the distribution of euchromatin and heterochromatin domains [69]. Forces acting upon chromosomes can thus modify transcriptional potential regardless of intermediate biochemical signaling pathways. Significant mechanosensitive transcriptional regulators include Yes-associated protein (YAP) and transcriptional coactivator with PDZ-binding motif (TAZ). Both YAP and TAZ are responsive to variations in cytoskeletal tension and substrate stiffness. Adequate mechanical stimulation enables YAP/TAZ to localize to the nucleus, bind TEAD transcription factors, and elicit gene expression programs regulating cellular growth, metabolic adaptations, and cytoskeletal organization [70]. Mechanical stimulation also modifies epigenetic machinery. Histone acetyltransferases (HATs), histone deacetylases (HDACs), and related chromatin-modifying enzymes adjust chromatin accessibility in response to mechanical loading through post-translational histone modifications. Enhanced activity-dependent histone acetylation has been linked to elevated transcription of genes controlling mitochondrial biogenesis, oxidative phosphorylation, and structural remodeling [71].

Therefore, mechanotransduction impacts higher-order regulatory systems that govern long-term cellular adaptation rather than merely initiating immediate signaling responses. Conversely, diminished mechanotransductive input progressively reduces the effectiveness of regulatory systems. Decreased cytoskeletal tension diminishes YAP/TAZ activation while also altering chromatin organization. Metabolic dysfunction and oxidative stress can also disrupt patterns of histone modification and DNA accessibility. Post-transcriptionally regulated noncoding RNA species, including microRNAs that regulate major anabolic/catabolic pathways, represent another layer of regulation impacted by the disease state [72].

Collectively, these mechanisms diminish skeletal muscle’s ability to dynamically regulate gene expression based upon fluctuations in environmental/physiological states. Specifically, loss of transcriptional plasticity may be very detrimental in patients with gastric cancer who experience fluctuations in metabolic demand, inflammatory burden, and treatment-induced stress necessitating rapid adaptive responses [73,74]. Overall, mechanotransduction can be considered a multi-scale signaling continuum spanning from sensing force outside the cell to regulating gene expression inside the cell. Gastric cancer appears able to disrupt this continuum at structural, ionic, metabolic, and transcriptional levels simultaneously. Consequently, disease progression may involve progressive reduction in mechano-structural continuity, wherein mechanical stimuli are no longer effectively translated into coherent biochemical/transcriptional responses. Failure of force-to-information translation may represent an underappreciated contributor to cancer-associated muscle wasting [75]. This area of study ties the primary title concept of mechanotransduction failure with muscle-wasting associated with gastric cancer. The systemically inflammatory environment, the oxidative state, poor nutrition, high treatment burden and low levels of physical activity in patients with gastric cancer could severely impair the skeletal muscles’ ability to generate a response through conversion of applied mechanical load into metabolic, transcriptional and anabolic responses. Thus this association underlies much of the basis for recommending that kinetotherapy should only be prescribed when there remains some level of responsiveness to mechanical loading of force-sensing cytoskeletal, ionic and nuclear pathways. Because mechanotransducing signaling is tightly linked to Ca^2+^ dynamics, the next section will focus on calcium-dysregulation as the link between mechanical-signal impairment; excitation-contracture failure; mitochondrial-stress; and proteolysis.

## 4. Calcium Dysregulation and Excitation–Metabolism Uncoupling

### 4.1. RyR1 Microdomain Instability, Post-Translational Remodeling, and Subcellular Ca^2+^ Leak Dynamics

The instability of the Ryanodine Receptor Type 1 (RyR1) microdomain itself, together with the post-translational remodeling of these regulatory complexes, may account for some of the functional loss observed in malignant diseases such as gastric cancer before significant changes in gene expression occur. Oxidative stress that accompanies many disease states may promote S-nitrosylation, S-glutathionylation, and carbonylation of RyR1. These modifications reduce the binding affinity of calstabin1 for RyR1 and increase the likelihood of spontaneous channel opening independent of membrane depolarization. Additionally, hyperphosphorylation of RyR1 by either PKA or CaMKII further destabilizes the RyR1/calstabin1 complex, resulting in a “leaky channel” phenotype characterized by low-level efflux of Ca^2+^ from the SR at rest [76,77,78].

SR Ca^2+^ leak does not occur evenly throughout the myofiber; instead, it appears to occur in well-defined subcellular microdomains. These microdomains create localized elevations in cytosolic Ca^2+^ that preferentially activate nearby signaling molecules, including redox-sensitive kinases, phosphatases, and transcriptional regulators, while having limited effects on the overall Ca^2+^ transient. Continuous SR Ca^2+^ leak results in progressive depletion of releasable SR Ca^2+^ stores, diminishes the strength of Ca^2+^ release during stimulation, and impairs the reliability of excitation–contraction coupling [79].

Additionally, diminished ATP availability may limit the capacity of sarco/endoplasmic reticulum Ca^2+^-ATPases (SERCA1 and SERCA2) to replenish Ca^2+^ in the SR. Sarcolipin (SLN) is another factor that contributes to this process by uncoupling SERCA ATP use from Ca^2+^ transport. This results in increased energy consumption without proportionally increased Ca^2+^ sequestration. Therefore, SR Ca^2+^ leak and defective SR Ca^2+^ reuptake form a positive feedback loop that progressively disrupts intracellular Ca^2+^ homeostasis [80].

Thus, unstable RyR1 microdomains and disrupted SERCA activity do not merely decrease the magnitude of Ca^2+^ signaling; they also distort its informational content. Instead of providing high-amplitude, physiologically relevant Ca^2+^ transients associated with activation, skeletal muscle may produce lower-amplitude stress-related Ca^2+^ oscillations similar to biological “noise.” This change may represent an initial phase in the progression from adaptive signaling to maladaptive remodeling and eventual muscle degeneration due to cancer-induced muscle wasting [81].

### 4.2. Store-Operated Ca^2+^ Entry, Mitochondrial Ca^2+^ Overload, and Metabolic Signal Distortion

Store-operated Ca^2+^ entry (SOCE) is critical for restoring depleted intracellular Ca^2+^ stores. SOCE commences once stromal interaction molecule 1 (STIM1) recognizes reduced luminal Ca^2+^ through its EF-hand sensor domains. Following detection, STIM1 undergoes a conformational change and dimerization and relocates to interfacial regions between the SR and plasma membrane. Here, STIM1 binds to Orai1 channels, establishing localized Ca^2+^ influx. Although SOCE is tightly regulated and generally transient under physiological conditions, it can persist for extended periods in the presence of chronic SR Ca^2+^ leak, particularly when driven by inflammation and oxidative stress accompanying gastric cancer [82].

Prolonged STIM1/Orai1 interaction facilitates prolonged cytoplasmic Ca^2+^ accumulation, particularly near mitochondria. Regions near mitochondria contain mitochondria-associated membranes (MAMs), which facilitate bidirectional transfer of ions, including Ca^2+^ [83]. Mitochondrial Ca^2+^ uptake via MAMs involves multiple molecular entities, including IP3 receptors (IP3Rs), VDACs, molecular chaperones, and the mitochondrial calcium uniporter (MCU). While moderate increases in mitochondrial Ca^2+^ stimulate activity of the TCA cycle and promote ATP synthesis, sustained cytoplasmic Ca^2+^ increases result in enhanced mitochondrial Ca^2+^ accumulation [84]. This leads to overstimulation of oxidative metabolism, higher ROS production, and destabilization of mitochondrial membrane potential. If this process progresses, it may lead to opening of the mPTP and subsequent collapse of the proton gradient, ATP depletion, and release of pro-apoptotic factors into the cytoplasm [85].

These mechanisms provide direct connections between calcium dysregulation and metabolic dysfunction. Instead of supporting efficient oxidative phosphorylation, excess mitochondrial Ca^2+^ loading leads to inefficient energy use, ROS generation, and chronic mitochondrial damage. ROS produced under these conditions may also enhance oxidation of RyR1 and SERCA proteins, thereby creating a positive feedback loop that enhances calcium dysregulation. Furthermore, disruptions in mitochondrial function lead to altered NAD^+^/NADH ratios, affecting sirtuin activity and modifying transcriptional responses to metabolic stress [86].

Activation of SOCE for prolonged periods also distorts the temporal resolution of intracellular signaling. High-fidelity rapid Ca^2+^ transients that are usually encoded with specific mechanical and electrical events are gradually replaced by slower, less precise cytoplasmic Ca^2+^ elevations. These signals preferentially activate stress-response pathways. This transition may help establish a disconnection between electrical/mechanical activation and coordinated metabolic response, referred to as excitation–metabolism decoupling [87].

### 4.3. Ca^2+^-Driven Proteolytic Convergence and Cytoskeletal Destabilization

High cytoplasmic Ca^2+^ concentrations promote activation of numerous proteolytic systems functioning in concert with ubiquitin–proteasome and autophagic pathways. Two such systems are µ-calpain (calpain-1) and m-calpain (calpain-2); however, their activation is dependent on cytoplasmic Ca^2+^ concentration, and thus they possess different activation thresholds. Upon activation, each proteolytically cleaves various structural proteins, including spectrin, talin, and vinculin, thereby disrupting the transmission of force between sarcomere units and the extracellular matrix [88].

Unlike ubiquitin-mediated proteolysis, calpain-mediated cleavage produces irreversible protein fragments that can modulate protein–protein interactions, disrupt cytoskeletal tension, and serve as substrates for additional proteolytic pathways. In parallel, calcium-dependent phospholipases break down membrane phospholipids, altering membrane fluidity and integrity. Such disruptions to structural proteins can also arise from unphysiological phosphorylation events [89].

Cytoplasmic Ca^2+^ may also initiate apoptosis. Elevated mitochondrial Ca^2+^ loading enhances the release of cytochrome c from mitochondria, facilitating formation of the apoptosome and initiation of executioner caspases. Crosstalk between calpains and caspases further exacerbates this process because calpain-mediated cleavage of members of the Bcl-2 family can facilitate release of pro-apoptotic factors from mitochondria [90]. Thus, elevated cytoplasmic Ca^2+^ serves as an upstream coordinator for multiple degradative pathways. The effect is not limited to protein degradation. Many calpain substrates are necessary for effective mechanotransduction. For example, calpain-mediated cleavage of costameric proteins reduces integrin-mediated attachment between sarcomeres and the extracellular matrix; likewise, disruption of cytoskeletal filaments impedes force transmission between sarcomeres. Both structural deficiencies negatively impact mechanotransductive pathways, reducing skeletal muscle’s ability to recognize, decode, and respond to mechanical stimuli [91]. Table 2 illustrates a concise overview illustrating how aberrant regulation of calcium handling diffuses across molecular/organelle/cellular scales relating micro-domain instability to metabolic dysfunction/structural damage to skeletal muscle.

As such, disruptions in calcium homeostasis must be considered as a primary mechanism connecting upstream redox/metabolic stress with downstream proteolytic/structural remodeling based on changes to both the amplitude and informational content of calcium signaling. As tightly regulated Ca^2+^ transients are replaced by longer-duration stress-related patterns, skeletal muscle transitions into a state of maladaptive remodeling across multiple biological levels. Ultimately, decreased signaling fidelity may provide one of the key mechanistic links between systemic stressors associated with gastric cancer and progressive declines in skeletal muscle structure, function, and adaptability observed in gastric cancer-induced muscle wasting [107]. This portion of this research study is also going to describe how disruptions in mechanotransduction can result in a failure of both structural and metabolic functions occurring in muscle atrophy resulting from gastric cancer. In this case, while Ca^2+^ primarily serves as a signaling molecule for contraction; it is a stress signifier that connects inflammation, oxidative injury, mitochondrial overload/dysfunction and an increase in proteolytic activity. Since coordinated excitation/contraction and excitation/metabolism couplings are required for successful kinesiotherapy, the next section of this research will examine the primary bioenergetic/redox interface in which disruption of Ca^2+^ levels, systemic inflammation and impaired muscle adaptation will be interdependent by describing the fragmentation of mitochondrial networks.

## 5. Mitochondrial Network Collapse and Redox–Immunometabolic Feedback

### 5.1. Electron Transport Chain Remodeling, Supercomplex Instability, and Redox Disequilibrium

Both the structure and function of mitochondria in skeletal muscle are dependent upon how the electron transport chain (ETC) is organized and how the different components of the chain interact. The electron transport chain includes complexes I–IV and ATP synthase, also known as complex V. These complexes work together to provide efficient electron transfer while minimizing unwanted electron leak. Efficiency in electron transfer is greatly affected by cardiolipin, a phospholipid found in the inner mitochondrial membrane. Cardiolipin helps stabilize larger respiratory structures made up of complexes I, III, and IV, thus supporting oxidative phosphorylation and reducing ROS overproduction [99]. During prolonged periods of systemic stress, as occurs in gastric cancer, this lipid–protein structure may become progressively unstable at lipid, protein, and supramolecular levels [108,109,110].

When cardiolipin is oxidatively modified, larger respiratory supercomplexes may become destabilized, electron-transfer rates may be altered, and electron leak may occur from complexes I and III. This leads to superoxide formation, followed by hydrogen peroxide formation and the creation of multiple reactive intermediates. Although low levels of ROS participate in tightly controlled signal transduction cascades, high levels of ROS can create redox imbalance in mitochondrial, cytoplasmic, and nuclear compartments through diffusion, secondary reactions, and activation of redox-sensitive pathways [111].

Post-translational oxidative modifications can reduce the functional capacity and efficiency of ETC subunits. These modifications can also disrupt protein–protein interactions needed to form stable supercomplexes. Additionally, certain chaperones that protect against protein oxidation are themselves susceptible to oxidation. If these chaperones become inactive, they may no longer protect newly synthesized mitochondrial proteins from aggregation or improper targeting during mitochondrial protein import. As a result, oxidative phosphorylation becomes less efficient, and proton-gradient formation becomes less effectively coupled to substrate oxidation [112].

The effects of ETC reorganization go beyond ATP production. High levels of ROS can activate redox-sensitive kinase, phosphatase, and transcription factor signaling pathways, thereby linking mitochondrial malfunction to broader cellular signaling responses. In gastric cancer, where inflammatory signaling is already present, mitochondria-generated ROS likely serves primarily to enhance existing inflammatory and catabolic signaling pathways rather than initiate them. Therefore, mitochondrial dysfunction can be viewed as a central component integrating metabolic and signaling disturbances resulting from systemic disease [113].

### 5.2. Suppression of Mitochondrial Biogenesis: AMPK–PGC-1α–SIRT1 Axis Decoupling and Transcriptional Inertia

In order for skeletal muscle mitochondria to operate properly, there needs to be both maintenance of the existing population of mitochondria and continued replacement through mitochondrial biogenesis. Both processes require coordinated signaling through AMPK, PGC-1α, and SIRT1. These regulatory elements stimulate transcription of genes encoding enzymes necessary for oxidative metabolism, mitochondrial DNA replication, respiratory-chain assembly, and metabolic adaptation [114].

AMPK is a major regulator of cellular energy status. It detects changes in the relative amounts of adenosine monophosphate (AMP) and adenosine triphosphate (ATP) within cells. Activation of AMPK results in phosphorylation of PGC-1α. Once PGC-1α is phosphorylated, it functions more effectively as a coactivator. Phosphorylated PGC-1α then forms complexes with transcription factors including NRF-1, NRF-2, and estrogen-related receptors (ERRs), stimulating transcription of genes necessary for mitochondrial DNA replication, transcription of the mtDNA genome, and oxidative phosphorylation. SIRT1 provides additional regulatory control by deacetylating PGC-1α using NAD^+^ as a cofactor. Deacetylation of PGC-1α links mitochondrial biogenesis to redox status and metabolic condition [115]. There are multiple ways in which gastric cancer can negatively affect this adaptive system. Systemic chronic inflammatory stimulation can inhibit AMPK activation through alteration of upstream kinases or disruption of the cellular mechanisms by which AMPK senses energy status. Oxidative stress can also lead to depletion of NAD^+^ pools. Reduced NAD^+^ availability decreases SIRT1’s ability to deacetylate PGC-1α. This further reduces the ability of PGC-1α to facilitate transcriptional activation. Overall, this may lead to a state of transcriptional inertia, whereby stimuli that typically induce mitochondrial biogenesis are unable to generate sufficient adaptive responses [116].

Reduced mitochondrial biogenesis may lead to accumulation of damaged mitochondria that cannot meet the demands placed upon them. This accumulation may increase ROS production. Decreased oxidative phosphorylation capacity, caused by reduced expression of mitochondrial proteins, may also contribute to reliance on less efficient metabolic pathways [117,118]. Impaired PGC-1α-mediated signaling may also affect the fiber-type distribution of skeletal muscle, leading to fibers that rely more heavily on glycolysis than aerobic metabolism. Ultimately, impaired PGC-1α signaling may make skeletal muscle less adaptable to changes in metabolic demand. Transcriptomic studies have identified gene expression profiles indicative of reduced mitochondrial biogenesis and diminished oxidative phosphorylation capacity. Collectively, these data indicate that mitochondrial biogenesis is not merely delayed but actively suppressed. Active suppression could represent a critical transition point from potentially reversible metabolic adaptation toward irreversible maladaptive reprogramming. Given that metabolic demands vary depending on treatment burden, nutritional status, and systemic inflammation associated with gastric cancer, failure to increase mitochondrial content may severely limit the capacity of skeletal muscle to adapt metabolically [119].

### 5.3. Mitochondrial Ca^2+^ Overload, Permeability Transition, and Self-Reinforcing Stress Networks

Calcium and metabolic signaling converge on mitochondria as both sensors and regulators of cellular homeostasis. Under physiological conditions, transient increases in cytosolic calcium are imported into mitochondria through the mitochondrial calcium uniporter (MCU). MCU activity is modulated by accessory proteins MICU1 and MICU2. While these proteins generally maintain appropriate calcium entry into mitochondria under resting conditions, they do not prevent moderate increases in cytosolic calcium from being imported into mitochondria. Importantly, moderate increases in cytosolic calcium stimulate key dehydrogenases in the TCA cycle. Consequently, cytosolic calcium signaling is directly connected to ATP production through calcium-stimulated TCA cycle activity [120].

However, when cytosolic calcium continues to rise above normal levels or is not properly recognized by mitochondria, mitochondrial calcium handling shifts away from adaptive regulation toward maladaptive overload. Elevated calcium entry into the mitochondrial matrix increases mitochondrial calcium loading and destabilizes the inner mitochondrial membrane. This increases the likelihood of opening of the mitochondrial permeability transition pore (mPTP), leading to dissipation of the proton gradient necessary for ATP synthesis, osmotic swelling, outer membrane rupture, release of pro-apoptotic proteins such as cytochrome c, and linking bioenergetic decline with apoptosis [121,122]. Elevated calcium loading within mitochondria also worsens oxidative stress. Increased calcium loading increases electron flow through the electron transport chain, increases electron leakage, and produces more ROS. ROS produced from elevated calcium loading can react with and modify mitochondrial proteins, lipids, and DNA, thereby sustaining mitochondrial dysfunction. ROS can also diffuse into the cytosol and modify upstream calcium-handling proteins such as RyR1 and sarco/endoplasmic reticulum Ca^2+^-ATPases (SERCA). The resultant positive feedback loop allows excessive calcium loading and redox imbalance to mutually reinforce each other [123].

Damage to mitochondria also participates in broader immunometabolic signaling. Damage-associated molecular pattern molecules released from damaged mitochondria can activate inflammatory and stress-response signaling pathways. For example, succinate can stabilize hypoxia-inducible factor alpha (HIF-α)-dependent signaling, whereas ROS produced from damaged mitochondria can activate inflammasome components and redox-sensitive transcription factors. In gastric cancer, where systemic inflammation already exists, mitochondria-produced signals may strengthen pre-existing inflammatory networks and integrate intracellular metabolic stress into systemic immune responses [124].

Therefore, excessive calcium loading, redox imbalance, and bioenergetic impairment converge into a self-reinforcing stress network. Within this network, mitochondrial dysfunction functions both as a consequence and as a driver of ongoing cellular injury. Interlocking feedback loops progressively decrease energetic efficiency, signaling accuracy, and structural stability. In this context, mitochondrial collapse constitutes a central intersection point coordinating excitation–contraction disturbances, metabolic disturbances, redox stress, and proteolytic activation, thereby supporting a significant role for mitochondrial dysfunction in the progression of skeletal muscle dysfunction in patients with gastric cancer [125]. To clarify the role of mitochondrial dysfunction within the broader model of gastric cancer-associated muscle wasting, Table 3 summarizes the main mitochondrial nodes discussed in this section.

This section has been designed to determine if the dysfunction of mitochondria alone is responsible for muscle wasting (cachexia) caused by cancer. Many components contribute to cachexia, such as; systemic inflammation, poor nutrition, treatment related side effects, increased intracellular Ca^2+^, and reduced mechanical activity contributing to the overproduction of ROS in the mitochondria, inefficient ATP production, and reduced ability of cells to adapt. Because these are potential contributors to the mitochondrial abnormalities, the effectiveness of kinetotherapy may also be impacted. In order for a kinetotherapy program to provide adaptive signaling and allow the body to begin adapting through the use of a new or modified movement pattern, there must be sufficient energy stores available and maintainable redox status. Thus, the focus of the next section will be on examining how proteostasis breaks down as a direct result of; mitochondrial stress, activation of inflammatory response pathways, imbalance in intracellular Ca^2+^ regulation, and impaired mechanotransduction.

## 6. Proteostasis Collapse: Ubiquitin–Proteasome, Autophagy, and Denervation-like Programs

### 6.1. Ubiquitin–Proteasome System Reprogramming: E3 Ligase Specificity, Substrate Hierarchy, and Signaling Feedback

Skeletal muscle proteostasis is selectively regulated toward protein degradation under systemic stress caused by chronic diseases, including gastric cancer. Importantly, this shift in proteolytic selectivity is not simply a reflection of overall increased proteolytic activity, but rather reflects a hierarchically structured selection of specific substrates for degradation. Different E3 ubiquitin ligases target different subsets of structural and regulatory proteins depending on the cellular environment. For example, MuRF1/TRIM63 and atrogin-1/FBXO32 specifically target and degrade structural and regulatory proteins based on cellular environmental signals. Specifically, MuRF1/TRIM63 primarily targets thick filaments, including myosin heavy chains and titin-associated proteins, thereby disrupting sarcomere structure and decreasing the ability of muscle to generate force. Atrogin-1/FBXO32 has a broader impact; it regulates the turnover rate of structural proteins and also modulates transcriptional coactivators, translation initiation factors, and signaling intermediary molecules [126].

Thus, atrogin-1/FBXO32 has an important role not only in regulating structural protein turnover, but also in regulating intramuscular signaling mechanisms related to mechanical adaptation and metabolic regulation. Substantial evidence indicates that ubiquitination during muscle atrophy is hierarchical. Anabolic signaling molecules may be ubiquitinated and degraded prior to significant loss of structural proteins [127]. Thus, this temporal hierarchy may limit cellular responsiveness before overt structural deterioration becomes apparent. Additionally, the type of ubiquitin linkage, particularly K48-linked versus K63-linked polyubiquitination, determines whether a molecule is directed to the proteasome for degradation or used as a scaffold for signaling. Thus, ubiquitin-chain topology provides another level of specificity in the regulation of proteostasis [128].

Both the ubiquitin–proteasome system and autophagy regulate signaling molecule levels through positive and negative feedback loops. Removal of upstream signaling molecules may increase catabolic responses by eliminating negative regulatory control. Additionally, degradation of proteins necessary for maintaining mitochondrial homeostasis or cytoskeletal organization may result in secondary effects on the metabolic and mechanotransductive properties of muscle. As such, activation of the ubiquitin–proteasome system acts as both an effector and a regulator of cellular reprogramming. Therefore, proteolysis should be viewed as an active participant in the reprogramming process rather than a final event [129].

### 6.2. Autophagy–Lysosome Flux Modulation: Selective Organelle Turnover and Metabolic Recalibration

Parallel to the ubiquitin–proteasome system, autophagy–lysosome flux provides a mechanism for the degradation of large protein aggregates and the selective removal of damaged organelles and macromolecules. Autophagy is an essential cellular process for maintaining cellular homeostasis and ensuring proper organelle quality control. Initiation of autophagy requires the formation of a complex composed of several proteins, including ULK1. This complex integrates signals related to nutrient availability, cellular energy status, and other forms of cellular stress. After activation by cellular signals, ULK1 promotes the development of phagophores, or pre-autophagosomal membranes. These membranes eventually develop into autophagosomes, which are able to sequester various types of cellular material. Finally, autophagosomes fuse with lysosomes and undergo degradation at low pH in an environment rich in hydrolytic enzymes [130].

Increased autophagy is often seen as a quantitative increase in autophagic activity. However, chronic systemic stress can lead to qualitatively distinct patterns of autophagic remodeling. Examples of selective forms of autophagy include mitophagy, which removes damaged mitochondria; reticulophagy, which removes damaged endoplasmic reticulum; and ribophagy, which removes damaged ribosomes. Mitophagy mediated by BNIP3, NIX, and PINK1/Parkin may initially protect cells by removing dysfunctional mitochondria [131]. However, prolonged mitophagic activity can ultimately lower total mitochondrial content and potentially reduce oxidative capacity. Recent studies suggest that autophagic flux can become decoupled from the efficiency of lysosomal degradative processes. This decoupling leads to accumulation of autophagic intermediates and therefore reduces clearance capacity. Abnormalities in lysosomal pH regulation, membrane permeability, or enzymatic activity can contribute to the accumulation of damaged proteins and organelles, thereby contributing to long-term functional decline [132].

**Figure 3:**
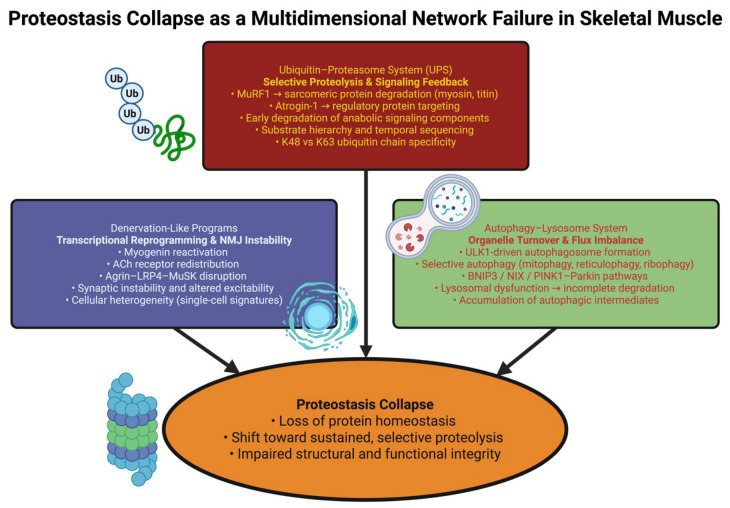
Proteostasis collapse in skeletal muscle is depicted as a multidimensional network failure arising from the convergence of three interdependent systems: ubiquitin–proteasome–mediated selective proteolysis, autophagy–lysosome–driven organelle turnover and flux imbalance, and denervation-like transcriptional reprogramming with neuromuscular junction instability. Their interaction reshapes signaling networks, impairs structural integrity, and drives a sustained shift toward catabolic remodeling.

Autophagy interacts directly with metabolic regulation through the recycling of amino acids, lipids, and nucleotides, thus providing substrates for subsequent metabolic pathways such as those controlled by mTOR and AMPK. In patients with gastric cancer, nutrient availability may be affected by tumor-derived metabolites, decreased food intake, treatment- or surgery-related side effects, and inflammation. Under these conditions, autophagic recycling may play an increasingly critical role in maintaining substrate availability. Conversely, overactive or unregulated autophagy results in a net loss of cellular material, thus promoting catabolic remodeling rather than regenerative recovery [133]. Figure 3 illustrates this relationship between ubiquitin–proteasome activity, autophagy–lysosome flux regulation, and denervation-like transcriptional programming as part of a model describing how these three interdependent systems converge to cause proteostasis collapse.

### 6.3. Denervation-like Transcriptional Reprogramming and Neuromuscular Junction Destabilization

A third factor that contributes to proteostasis collapse is denervation-like transcriptional reprogramming without overt neuronal damage. Denervation-like transcriptional reprogramming refers to widespread reorganization of gene expression involving diminished synaptic stability, altered excitability, and increased susceptibility to proteolytic degradation. One characteristic feature of this process is enhanced activation or repression of developmental transcription factors, including myogenin, that regulate genes involved in neuromuscular remodeling and muscle atrophy [134].

Denervation-like transcriptional reprogramming is associated with enhanced mRNA levels encoding α- and β-subunits of acetylcholine receptors on the surface of skeletal muscle fibers. It is also associated with changes in acetylcholine receptor localization and distribution, as well as disruption of agrin/LRP4/MuSK signaling pathways, leading to synaptic instability. Collectively, these changes may contribute to reduced synaptic efficacy, disrupted excitation–contraction coupling, and impaired function [135]. Many changes associated with denervation-like transcriptional reprogramming occur simultaneously with enhanced degradative pathways. This indicates that destabilized neuromuscular junctions and protein degradation represent coordinated aspects of an overarching adaptive response rather than isolated occurrences. Studies using single-cell and single-nucleus RNA sequencing demonstrate that chronic systemic stress causes marked heterogeneity among cells within skeletal muscle, with some populations demonstrating transcriptional profiles associated with denervation-related metabolic dysfunction and stress adaptation [136,137].

Collectively, these data indicate that proteostasis failure is structurally and functionally heterogeneous, with local areas within muscle exhibiting unique patterns of disrupted signaling and remodeling. These findings support the notion that proteostasis failure represents a multilayered disruption in protein quality control, organelle turnover, and adaptive gene-expression programs. Under normal conditions, these systems work together to maintain proteostasis and support muscle plasticity. However, under conditions analogous to gastric cancer-induced muscle wasting, they progressively shift away from homeostatic regulation and move toward catabolic remodeling rather than regenerative repair. Proteostasis collapse may therefore be viewed as a multidimensional failure of proteomic governance, contributing to continued decline in structural integrity, metabolic adaptability, and functional capacity [138]. Therefore, proteostasis failure represents the downstream focal point of convergence for all the upstream pathways described above as it relates to gastric cancer-induced muscle wasting. As such, through their combined effects (i.e., inflammatory signaling; SMAD activation via myostatin–ACVR2B; mTOR/Akt suppression; diminished mechanotransduction; Ca^2+^ deregulation; and mitochondrial dysfunction), these signals push skeletal muscle towards continued degradation rather than restoration. Given that skeletal muscle can only be supported by the physical stimulation provided during structured kinetic therapy, if skeletal muscle has lost its ability to perform the necessary proteolytic functions to restore damaged or degraded proteins, maintain organellar quality, and maintain neuromuscular function then there is no potential for structural or functional recovery. Therefore, the focus will shift in the next section to describe how systemic kinetic interventions may be used to restore coordinated biological responses rather than merely targeting individual biochemical pathways.

## 7. Molecular Reprogramming by Kinetotherapy: Reversing the Catabolic Network

### 7.1. Recalibration of Inflammatory–Catabolic Circuits: Attenuation of IL-6/STAT3 and NF-κB Signaling Topology

Structured mechanical inputs (e.g., Kinetotherapy) can modulate inflammatory signaling in a manner consistent with “recalibrating” rather than completely suppressing inflammatory signals. The degree to which kinetotherapy reduces or abolishes the inflammatory response is less important than how kinetotherapy alters the regulatory parameters of those responses. For example, repeated activation of skeletal muscle by mechanically induced contraction results in a cytokine response that is both transient (as opposed to chronic) and highly regulated. The nature of this response is distinct from the chronic, low-grade inflammatory state typically observed in malignant disease. Therefore, the repetitive nature of the mechanical stimulus applied to the skeletal muscle may disrupt the persistent downstream transcriptional program generated by continuous activation of STAT3 and NF-κB [139,140].

The intracellular signaling events initiated by contraction-induced signaling include the enhancement of phosphatase activity (e.g., SHP2 and PP2A), which results in the dephosphorylation of STAT3 at Tyr705 and reduction of its nuclear localization. Concurrently, modulation of the cellular redox environment (through enhanced mitochondrial efficiency and/or increased cellular antioxidant defense) may limit the activation of the IKK complex resulting in decreased phosphorylation/degradation of IκB and subsequent reduction in NF-κB’s nuclear translocation. However, these actions do not eliminate the generation of inflammatory signals; instead they facilitate the transition from a sustained self-regulatory pattern of inflammatory signaling towards a more transient regulated pattern of signaling [141,142].

Exercise-generated secretions of anti-inflammatory mediators (such as IL-10 and specific myokines) could further modulate systemic immunological tone. These anti-inflammatory mediators can regulate macrophage polarization. Specifically, these anti-inflammatory mediators would favor macrophage phenotypes associated with resolving inflammation rather than propagating it [143]. Thus, such alterations may be particularly significant in gastric cancer where immune signaling operates at the nexus between host adaptability and tumor biology [144]. Observations from clinical studies involving preoperative exercise interventions in patients undergoing surgery for gastric cancer demonstrate a decrease in postoperative inflammatory markers along with faster recovery rates. Collectively, these findings indicate that the modulation of inflammatory signaling can result in observable physiological outcomes [145].

More importantly, this modulation may be viewed as a change in network dynamics (i.e., feedback loops and signal persistence) rather than a uni-directional inhibition. Through its action on network dynamics, kinetotherapy will likely decrease the likelihood that inflammatory pathways remain in a high-state activity pattern thereby indirectly affecting downstream processes such as proteolysis, mitochondrial stress and metabolic dysfunction [146]. To clarify how this inflammatory recalibration may operate in gastric cancer-associated muscle wasting, Table 4 summarizes the main kinetotherapy-related inputs, their inflammatory targets, expected recalibrating effects, and their relevance to muscle preservation.

### 7.2. Reconstitution of Mechanotransductive Fidelity and Normalization of Calcium Signaling Architecture

One of the primary molecular interfaces through which kinetotherapy can produce its effects is through restoring mechanotransductive signaling. Repetitive mechanical loading engages costamere-integrin complexes re-establishes focal adhesion structure assembly and increases the efficacy with which mechanical forces are converted to biochemical signals. This restoration of continuity between mechanical force application and biochemical response will increase the rate at which mechanical stimuli are converted to adaptive signaling [147].

At the level of ion channel-mediated mechanosensation, kinetotherapy induces reproducible patterns of Ca^2+^ influx through mechanosensitive channels (e.g., Piezo1 and TRP). These Ca^2+^ transients have defined amplitudes and frequencies. Thus, kinetotherapy may offset aberrant low-amplitude Ca^2+^ fluxes present in many pathological states. Restoration of normal Ca^2+^ signaling may also allow for reactivation of downstream effectors such as CaMKII and calcineurin enabling the reactivation of activity dependent transcriptional programs that enable metabolic and structural adaptations [148].

Mechanical stimulation may also impact the stability and regulation of proteins responsible for handling calcium. Studies conducted outside of oncologic contexts have demonstrated that exercise can decrease pathological leakiness of RyR1 possibly through improvement in redox status and restoration of protein interactions that stabilize RyR1. Exercise has also been shown to enhance SERCA activity which is indicative of enhanced Ca^2+^ uptake efficiency leading to the restoration of intracellular Ca^2+^ gradient and dynamic range. Although direct evidence for these mechanisms in gastric cancer are lacking, they represent a clear conceptual framework through which kinetotherapy can re-establish excitation-metabolism coupling [149].

Finally, restoration of cytoskeletal tension and mechanotransduction may activate mechanosensitive transcriptional regulators (YAP and TAZ) allowing them to interact with TEAD transcription factors and reactivate transcriptional programs related to cytoskeleton organization, mitochondrial function and metabolic adaptation. Consequently, kinetotherapy does not simply restore damaged signaling pathways but enables sustained transcriptional plasticity necessary for long-term adaptation [150].

### 7.3. Reactivation of Anabolic and Metabolic Integration: Akt/mTOR Signaling, Mitochondrial Renewal, and Systemic Metabolic Alignment

The convergence of enhanced mechanotransduction, restored calcium signaling, and diminished inflammatory burden create conditions favorable for the activation of anabolic metabolic pathways. Mechanical loading activates the PI3K/Akt/mTOR pathway via several mechanisms including integrin-associated signaling, phosphatidic acid production and cross-talk with growth factor activated pathways. As a consequence, mTORC1 target proteins such as p70S6 kinase and 4EBP1 are phosphorylated stimulating translation initiation and increasing ribosome activity [151]. Although some portion of anabolic signaling competency is likely irreversibly lost due to prolonged exposure to catabolic environments in gastric cancer patients; kinetotherapy may be sufficient to shift the equilibrium toward a condition in which net protein synthesis exceeds net protein degradation. The ability to achieve this partial restoration of anabolic competency may be particularly advantageous in gastric cancer given the reduced sensitivity exhibited by muscle tissue to nutritional/hormonal stimuli [152].

Enhanced mitochondrial function, achieved through activation of AMPK during exercise followed by coordinated induction of PGC-1α, can stimulate mitochondrial biogenesis and enhance oxidative capacity. Enhanced oxidative capacity will lead to decreased ROS production, enhanced ATP availability and re-established coupling between energy requirements and availability. Furthermore, kinetotherapy may induce enhancements in substrate utilization creating greater metabolic flexibility allowing for more efficient transitions between lipid/glucose oxidation [153]. Improvements in insulin signaling have also been reported after kinetotherapy. Specifically, enhanced phosphorylation of IRSs as well as increased translocation of GLUT4 to the sarcolemma have been noted. Insulin resistance may be exaggerated in gastric cancer patients secondary to surgical alteration in nutrient delivery and systemic inflammation. Stabilization of metabolic homeostasis through enhancement of insulin signaling may therefore be beneficial in gastric cancer patients [154].

Emerging evidence suggests that physical activity influences composition of the gut microbiome as well as short chain fatty acid production thereby establishing potential links between peripheral metabolic improvements and broader systemic effects. Collectively these mechanisms establish that kinetotherapy functions as a convergent regulator/modulator of multiple disrupted pathways rather than being solely focused upon inhibiting one specific mechanism [155]. Through simultaneous effects upon inflammatory signaling pathways, mechanotransduction, calcium dynamics, anabolic pathways and mitochondrial function, kinetotherapy may facilitate a restorative process regarding signaling coherency across multiple organizational levels [156].

## 8. Conclusion: Toward a Systems-Level Reinterpretation of Kinetotherapy in Gastric Cancer

Muscle dysfunction associated with gastric cancer, while manifesting as a catabolic condition, appears to reflect a more fundamental disruption of the biological integration of regulatory architectures operating at various scales. Thus, muscle loss and functional decline may result from the disruption of synchronized communication among previously intact signaling networks (mechanotransduction, calcium homeostasis, mitochondrial energetics, proteostasis, and transcriptional plasticity). As such, muscle cannot simply be viewed as mass or a force-generating entity. Rather, muscle may be thought of as an adaptive biological system that responds to physiological signals. When this capacity is compromised, so too are the abilities to recover from physiological insult and to respond effectively to external stimuli (therapeutic, environmental, etc.).

Given this new perspective on muscle and muscle dysfunction, one might consider revisiting the use of kinetotherapy. Traditionally, kinetotherapy has been utilized as a method to re-establish physical performance abilities. However, given its potential for acting as a structured physiological stimulus that can interact with distributed regulatory networks at various levels of biological organization, kinetotherapy may be seen as a therapeutic modality capable of engaging biological mechanisms that are typically resistant to modification by single-agent pharmacologic interventions. Additionally, because many of the pathways engaged by mechanical loading are similar to those involved in muscle dysfunction secondary to gastric cancer, kinetotherapy may directly engage the affected biological networks rather than serve only as a supportive intervention. In doing so, kinetotherapy’s role in supporting functional recovery could expand to that of a modality capable of inducing changes in the regulation of dysfunctional networks.

Additionally, it should be acknowledged that responses to kinetotherapy will likely vary significantly among individuals. The degree of benefit provided by kinetotherapy will likely depend upon a variety of factors, including pre-existing metabolic reserves, the level of systemic inflammation and oxidative stress present, nutritional status, the treatment burden experienced, and the timing of kinetotherapy initiation relative to other forms of therapy received (surgical and oncologic). The variation observed in response to kinetotherapy illustrates some of the limitations inherent in utilizing universally standardized approaches to rehabilitation. This variability highlights the need for rehabilitation programs for cancer patients that are based on more adaptable, individualized, and biologically informed principles. Importantly, the effects of kinetotherapy may extend beyond localized muscle physiology. Skeletal muscle serves as a metabolically active organ capable of producing endocrine-related signals (e.g., myokines), immune-related signals (e.g., cytokine production), and metabolic signals (e.g., substrate utilization patterns); therefore, mechanical stimulation may affect not only the structure and function of muscle but also the broader physiological processes listed above. While the full scope of systemic effects produced by exercise-based interventions remains poorly understood, mounting evidence suggests that they may exert effects that extend beyond the bounds of traditional rehabilitative practice.

As mentioned earlier, this review was designed to provide an integrated model that links the molecular mechanisms responsible for skeletal muscle dysfunction associated with gastric cancer with the systems-level effects of kinetotherapy. While there exists considerable experimental evidence demonstrating that many of these interactions occur across diverse biological systems, it is highly unlikely that each interaction occurs similarly in every patient with gastric cancer. Rather, each interaction is likely influenced by numerous interdependent relationships among genetic, metabolic, immunological, environmental, and clinical variables. While this complexity presents challenges in developing predictive models for therapeutic outcomes, it also affords opportunities to identify novel targets for mechanism-based modulation and to personalize rehabilitation strategies. Emerging technologies such as multi-omics analysis, spatial biology, digital phenotyping, and systems-level computational modeling may provide useful tools for identifying the biological states that are most responsive to mechanical stimulation.

Furthermore, it is essential to recognize the inherently dynamic nature of the biological processes described throughout this review. The progression from adaptive responses to increasingly entrenched dysfunction is unlikely to occur suddenly; instead, the transition is likely to be characterized by a continuous process driven by repetitive exposure to cumulative physiological stressors and self-reinforcing feedback loops. Therapies capable of interacting with this continuum and modifying system behavior rather than attempting to reverse it abruptly may provide unique advantages. In this regard, kinetotherapy may demonstrate how sustained exposure to low-intensity physiological stimuli can influence the behavior of complex adaptive systems under physiologically relevant constraints. In addition to being a tissue vulnerable to injury during gastric cancer, skeletal muscle may represent a biological interface through which systemic resilience can be maintained. Further research is needed before definitive therapeutic applications can be established. However, the alignment between pathways affected by mechanical stimulation and those altered during gastric cancer-associated muscle dysfunction provides a biologically plausible rationale for continued investigation. Therefore, the possibility that kinetotherapy can preserve physiological adaptability and contribute to systemic resilience during gastric cancer warrants continued examination.

The intent of this review was to develop an integrated conceptual framework that expands our understanding of skeletal muscle dysfunction in gastric cancer and highlights the potential role of mechanobiological interventions in its management. If this review assists in clarifying current paradigms, promotes cross-disciplinary dialogue, or stimulates further investigation into the systems-level effects of kinetotherapy in oncology, then it will have accomplished its goal.

## Figures and Tables

**Table 1:** Integrates the principal signaling axes governing cancer-associated muscle wasting, mapping ligand–receptor activation through intracellular cascades to net anabolic suppression and proteolytic activation. It highlights key convergence points (SMAD–Akt–FoxO), minimal measurable readouts, and actionable nodes, providing a compact framework for interpreting pathway imbalance and targeting restoration of muscle homeostasis.

Biological Module	Core Mechanism	Relevance to Muscle Wasting	Representative Readouts	Intervention Relevance	References
Myostatin–activin signaling	Myostatin/activin → ACVR2B–ALK4/5 → SMAD2/3 phosphorylation → SMAD4 nuclear signaling	Suppresses myogenesis and limits anabolic responsiveness	p-SMAD2/3; reduced myogenic gene expression; anabolic resistance markers	ACVR2B blockade; anti-myostatin/activin strategies; anabolic stimulation	[38]
Tumor–host endocrine amplification	Tumor- and host-derived IL-6, IL-8, and FSTL-1 sustain cytokine/ligand availability	Maintains chronic catabolic and SMAD-associated signaling	Circulating cytokines; portal/peripheral gradients; inflammatory pathway activity	Anti-inflammatory strategies; tumor–host loop disruption; inflammatory monitoring	[39]
Vascular–metabolic support	Activin A-related signaling reduces PGC-1α activity and impairs endothelial function	Limits perfusion, oxygen delivery, and metabolic support	Capillary density; endothelial markers; oxidative capacity; PGC-1α readouts	Kinesitherapy/exercise-based vascular stimulation; metabolic/endothelial support	[40]
Basal anabolic signaling	IRS-1 → PI3K → Akt → mTORC1 → p70S6K and 4E-BP1 regulation	Maintains translational capacity and protein synthesis	p-Akt Thr308/Ser473; p-p70S6K; 4E-BP1 status; protein synthesis markers	Nutritional optimization plus mechanical activation; anabolic restoration	[41]
SMAD–Akt interference	SMAD2/3 promotes IRS-1 degradation, phosphatase activation, and reduced Akt signaling	Weakens anabolic recovery after systemic stress	IRS-1 abundance; reduced p-Akt; impaired mTORC1 signaling	SMAD–Akt decoupling; Akt reactivation; combined anabolic/anti-catabolic therapy	[42]
Translational repression	Reduced mTORC1 activity lowers p70S6K activation and increases 4E-BP1 restriction of eIF4E	Limits translation initiation and ribosomal output	eIF4E availability; 4E-BP1 binding; p70S6K activity; translation rate	mTOR sensitization; nutritional, hormonal, and mechanical stimulation	[43]
FoxO-dependent catabolic transcription	Reduced Akt permits FoxO dephosphorylation and nuclear translocation	Activates proteolysis, autophagy, and atrophy programs	Nuclear FoxO1/3; FoxO target genes; catabolic transcriptional signatures	Akt restoration; FoxO modulation; anti-catabolic intervention	[44]
Ubiquitin–proteasome degradation	MuRF1/TRIM63 and atrogin-1/FBXO32 promote ubiquitination and proteasomal degradation	Accelerates sarcomeric breakdown and structural protein loss	MuRF1/TRIM63; FBXO32; ubiquitinated protein burden; proteasome activity	E3 ligase targeting; proteasome modulation; sarcomere preservation	[45]
Autophagy–mitophagy imbalance	ULK1, LC3, and BNIP3 regulate autophagosome formation and mitochondrial turnover	Supports quality control initially, but persistent activation may cause organelle loss	LC3-II; BNIP3; mitophagy markers; autophagic flux	Autophagy tuning; mitochondrial support; prevention of excessive organelle depletion	[46]
SMAD–FoxO convergence	SMAD transcriptional repression and FoxO activation converge with reduced Akt/mTOR signaling	Links anabolic suppression with proteolytic amplification	SMAD/FoxO target-gene co-expression; combined pathway activity scores	Dual-pathway inhibition; anabolic support plus catabolic suppression	[47]
Catabolic priming	Persistent FoxO activation with low Akt/mTOR baseline lowers the degradation threshold	Produces poor anabolic response and high stress sensitivity	Basal proteolysis markers; reduced anabolic response; persistent catabolic genes	Early integrated anabolic and anti-catabolic therapy	[48]
Patient-specific heterogeneity	Variable mTOR suppression, fiber-type remodeling, and metabolic shifts define distinct molecular profiles	Explains divergent wasting trajectories and treatment responsiveness	Transcriptomic subtypes; fiber-type markers; metabolic pathway scores; signaling profiles	Stratified intervention design; personalized therapy; selection for kinesitherapy/targeted strategies	[49]

**Table 2:** Summarizes calcium dysregulation as a linked pathological cascade in which RyR1 microdomain instability and SERCA inefficiency convert tightly regulated calcium transients into persistent low-amplitude leak signals, promoting chronic store depletion, sustained SOCE activation, and biased calcium transfer to mitochondria. This remodeling progressively shifts calcium from a contractile messenger to a stress signal, driving mitochondrial oxidative inefficiency, redox-amplified channel dysfunction, calpain- and caspase-linked proteolysis, cytoskeletal destabilization, and ultimately excitation–metabolism uncoupling within skeletal muscle.

Regulatory Node	Core Molecular Event	Signaling Effect	Functional Consequence	References
RyR1 instability	Oxidation, nitrosylation, hyperphosphorylation, and FKBP12 loss	Resting SR Ca^2+^ leak	Reduced contractile fidelity; increased stress signaling	[92]
Leaky release units	Persistent low-level RyR1 channel opening	Ca^2+^ noise replaces pulsatile signaling	Reduced excitation–contraction precision	[93,94]
SERCA impairment	ATP deficit, oxidative damage, and sarcolipin-mediated uncoupling	Impaired Ca^2+^ reuptake	Increased resting cytosolic Ca^2+^ and energetic cost	[95]
SR handling imbalance	Combined Ca^2+^ leak and weak resequestration	Lower transient amplitude and reduced dynamic range	Maladaptive Ca^2+^ encoding	[96]
Persistent SOCE	STIM1 clustering and sustained Orai1 opening	Continuous Ca^2+^ entry	Junctional Ca^2+^ accumulation near mitochondria	[83,97]
Mitochondrial Ca^2+^ overload	Excess MCU-mediated Ca^2+^ uptake	ROS generation and membrane depolarization	ATP inefficiency and oxidative stress	[98]
MAM flux amplification	IP3R–VDAC-mediated Ca^2+^ transfer	Spatially biased Ca^2+^ propagation	SR–mitochondrial stress coupling	[84]
ROS feedback loop	ROS-mediated RyR1 and SERCA remodeling	Increased leak and pump failure	Self-amplifying Ca^2+^ instability	[99,100]
Metabolic distortion	NAD^+^/NADH shift and altered sirtuin signaling	Stress-biased metabolic signaling	Reduced adaptive flexibility	[101]
Calpain activation	Ca^2+^-dependent protease activation	Rapid structural protein cleavage	Costamere and sarcomere destabilization	[102]
Caspase recruitment	Mitochondrial permeabilization and cytochrome c release	Apoptotic proteolysis	Proteolytic amplification	[103]
Membrane–cytoskeletal damage	Phospholipase activation and filament cleavage	Loss of tension and membrane integrity	Mechanotransduction failure	[104]
Proteolytic convergence	Calpain, caspase, UPS, and autophagy crosstalk	Net degradation bias	Progressive muscle wasting	[105]
Excitation–metabolism uncoupling	Persistent Ca^2+^ noise with mitochondrial inefficiency	Mechanical output no longer matches ATP production	Fatigue, weakness, and persistent dysfunction	[106]

**Table 3:** Mitochondrial network collapse in gastric cancer-associated muscle wasting. This table summarizes how mitochondrial dysfunction connects redox stress, calcium overload, impaired bioenergetics, inflammatory amplification, and reduced adaptive capacity in gastric cancer-associated muscle wasting.

Mitochondrial Node	Core Disturbance	Downstream Effect	Relevance to Gastric Cancer-Associated Muscle Wasting	Link to Kinetotherapy
ETC supercomplex instability	Cardiolipin oxidation and disrupted organization of complexes I, III, and IV	Electron leak and increased ROS production	Amplifies inflammatory and catabolic signaling already present in gastric cancer	Improved mitochondrial efficiency may reduce chronic redox stress
Redox disequilibrium	Excess mitochondrial ROS in mitochondrial, cytosolic, and nuclear compartments	Activation of redox-sensitive kinases and transcription factors	Reinforces proteolysis, inflammation, and impaired anabolic responsiveness	Repeated therapeutic movement may support antioxidant defenses
Impaired mitochondrial protein maintenance	Oxidative damage to ETC subunits and chaperone dysfunction	Reduced oxidative phosphorylation efficiency	Limits ATP availability for repair, contraction, and adaptive remodeling	Better energy efficiency may improve tolerance to rehabilitation
AMPK–PGC-1α–SIRT1 suppression	Reduced AMPK activation, NAD^+^ depletion, and impaired PGC-1α activity	Suppressed mitochondrial biogenesis and transcriptional inertia	Prevents replacement of damaged mitochondria and reduces oxidative capacity	Kinetotherapy may stimulate mitochondrial renewal through AMPK–PGC-1α signaling
Metabolic inflexibility	Reduced oxidative capacity and greater reliance on inefficient substrate use	Poor matching between energy demand and ATP production	Contributes to fatigue, weakness, and reduced functional reserve	Structured exercise may improve substrate utilization and metabolic flexibility
Mitochondrial Ca^2+^ overload	Excess MCU-mediated Ca^2+^ uptake and mPTP opening	Membrane depolarization, ATP depletion, and apoptosis-linked signaling	Links calcium dysregulation with bioenergetic collapse and muscle loss	Improved Ca^2+^ handling may support excitation–metabolism coupling
SR–mitochondrial redox feedback	ROS-mediated RyR1 and SERCA dysfunction	Further Ca^2+^ leak, impaired reuptake, and redox amplification	Creates a self-reinforcing cycle of calcium and mitochondrial instability	Redox stabilization may help preserve calcium-handling efficiency
Immunometabolic signaling	Release of mitochondrial stress signals, ROS, and metabolic intermediates	Activation of inflammatory and stress-response pathways	Strengthens systemic inflammation and catabolic remodeling in gastric cancer	Kinetotherapy may help shift systemic signaling toward adaptive regulation
Bioenergetic failure	Reduced ATP production and inefficient oxidative phosphorylation	Impaired contraction, repair, and protein-quality control	Lowers skeletal muscle resilience during cancer treatment and recovery	Adequate energetic reserve is necessary for effective rehabilitation adaptation

**Table 4:** Kinetotherapy-mediated recalibration of inflammatory–catabolic signaling in gastric cancer-associated muscle wasting. This table summarizes how structured therapeutic movement may shift persistent IL-6/STAT3 and NF-κB activation toward more transient, regulated, and adaptive inflammatory signaling, thereby reducing downstream catabolic amplification in gastric cancer-associated muscle wasting.

Kinetotherapy-Related Input	Main Inflammatory Target	Recalibrating Effect	Relevance to Gastric Cancer-Associated Muscle Wasting
Repeated supervised muscle contraction	Chronic IL-6/STAT3 signaling	Shifts cytokine signaling from persistent activation toward transient contraction-associated responses	May reduce sustained STAT3-driven catabolic transcription and improve adaptive muscle responsiveness
Mechanical activation of skeletal muscle	STAT3 Tyr705 phosphorylation	Supports reduced STAT3 phosphorylation and nuclear localization through phosphatase-associated mechanisms	May weaken inflammatory amplification of proteolysis and anabolic resistance
Improved redox control during repeated movement	IKK–IκB–NF-κB pathway	Limits excessive NF-κB activation by reducing redox-sensitive inflammatory signaling	May decrease persistent inflammatory and proteolytic signaling in cachectic skeletal muscle
Exercise-induced myokine response	Systemic immune tone	Promotes anti-inflammatory and inflammation-resolving mediator patterns	May counteract chronic low-grade inflammation associated with gastric cancer and treatment burden
Modulation of macrophage activity	Innate immune phenotype	Favors resolution-associated rather than propagation-associated inflammatory states	May reduce local and systemic inflammatory pressure contributing to muscle catabolism
Preoperative or perioperative exercise exposure	Surgical inflammatory response	May reduce postoperative inflammatory burden and support recovery dynamics	May improve resilience during gastric cancer surgery and perioperative stress
Repeated low-to-moderate physiological stimulation	Inflammatory feedback loops	Reduces signal persistence rather than abolishing immune signaling	May prevent inflammatory pathways from remaining locked in a high-activity catabolic state
Network-level inflammatory recalibration	Proteolysis, mitochondrial stress, and metabolic dysfunction	Indirectly weakens downstream catabolic amplification across interconnected pathways	Supports kinetotherapy as a systems-level intervention rather than a single-pathway treatment

## Data Availability

No new data were created or analyzed in this study. Data sharing is not applicable to this article.
